# Mimicking Insect Communication: Release and Detection of Pheromone, Biosynthesized by an Alcohol Acetyl Transferase Immobilized in a Microreactor

**DOI:** 10.1371/journal.pone.0047751

**Published:** 2012-11-14

**Authors:** Lourdes Muñoz, Nikolay Dimov, Gerard Carot-Sans, Wojciech P. Bula, Angel Guerrero, Han J. G. E. Gardeniers

**Affiliations:** 1 Department of Biological Chemistry and Molecular Modeling, IQAC (CSIC), Barcelona, Spain; 2 Mesoscale Chemical Systems, MESA+ Institute for Nanotechnology, University of Twente, Enschede, The Netherlands; INRA-UPMC, France

## Abstract

Infochemical production, release and detection of *(Z,E)*-9,11-tetradecadienyl acetate, the major component of the pheromone of the moth *Spodoptera littoralis*, is achieved in a novel microfluidic system designed to mimic the final step of the pheromone biosynthesis by immobilized recombinant alcohol acetyl transferase. The microfluidic system is part of an “artificial gland”, i.e., a chemoemitter that comprises a microreactor connected to a microevaporator and is able to produce and release a pre-defined amount of the major component of the pheromone from the corresponding *(Z,E)*-9,11-tetradecadienol. Performance of the entire chemoemitter has been assessed in electrophysiological and behavioral experiments. Electroantennographic depolarizations of the pheromone produced by the chemoemitter were ca. 40% relative to that evoked by the synthetic pheromone. In a wind tunnel, the pheromone released from the evaporator elicited on males a similar attraction behavior as 3 virgin females in most of the parameters considered.

## Introduction

Semiochemicals are chemical compounds that allow the transfer of information between species. These compounds are produced by one individual, i.e. the emitter, to elicit a behavioral and/or physiological response in another individual or group of individuals, i.e. the receiver. Often, the semiochemicals, which typically are molecules with relatively short chain-lengths and low molecular weight, are transported within a stream of air. Pheromones are a class of semiochemicals that trigger an individual or group of individuals from the same species and are used for behavioral responses such as trail or territory marking, alarm, synchronization, species aggregation, or mate attraction [1]–[4]. Interest in insect pheromone communication in the past has been related mostly to practical application in pest control in agricultural production [5], recently the focus is more on the fundamental understanding of pheromone communication, such as olfactory receptor response to a pheromone blend of a specific composition and with a specific spatiotemporal pattern and subsequent neurological mechanisms of decoding of the signals (see e.g. [6], [7]). As Olsson and coworkers point out [8], to gain more insight into the neurological responses of insects to semiochemicals, novel technological platforms for olfactory experiments and dissipation of volatiles are required, which is one of the underlying aims of the research described in this contribution.

The mechanisms for synthesis and dissipation occurring in nature may become inspirational in the design of such tools. For example, in moths, release to the environment of specific pheromone blends is generally carried out by females and emitted molecules are detected by males with a highly sensitive olfactory system. *In vivo* these blends are biosynthesized in a pheromone gland in most cases from fatty acids by pheromone specific enzymes [9]. As an example of the structure of a gland, the gland of the leiodid beetle, *Speonomous hydrophilus*, has a porous plate consisting of an epicuticular layer perforated by tiny pores, located at the opening of the gland [10]. Another interesting example of pheromone dissipation is that in which a female insect secretes sex pheromone onto hairs on her thorax, from which they evaporate into a wind stream [11]. We specifically mention these two examples, because they may be mimicked in a straightforward manner by state-of-the-art microfabrication technology, as will be demonstrated in this paper.

In a novel approach to information and communication technology, we have developed a chemoemitter system consisting of a microreactor connected to a microevaporator, capable to continuously produce and release a pre-defined amount of pheromone compounds. In this context, we report herein the infochemical production of (*Z,E)*-9,11-tetradecadienyl acetate (in short (*Z,E*)-9,11-14:OAc), the major component of the pheromone of the Egyptian armyworm *S. littoralis* (Lepidoptera: Noctuidae) [12], from the corresponding alcohol (*Z,E*)-9,11-tetradecadienol (in short (*Z,E*)-9,11-14:OH) by an alcohol acetyl transferase (*atf*), mimicking the last step of the pheromone biosynthesis inside the microreactor.

Using concepts of compartmentalization and integration, different microfluidic modules each performing one specific enzymatic reaction step (or separation step, if required) of a multienzyme sequence could in principle perform the entire biosynthetic pathway of insect pheromones, starting from similar ingredients as used in the real biological system. An illustrative example of the possibilities was recently given in a study by Lee et al., who designed and implemented a microreactor that was capable of biocatalysis in three consecutive steps: invertase, glucose oxidase, and soybean peroxidase were used in a sequence to yield H_2_O_2_ from glucose [13]. However, for the case of pheromones we have found that isolating the required enzymes in a stable form is an enormous task, which in the end forced us to restrict the artificial biosynthetic system to just one enzyme, namely an alcohol acetyl transferase.

In the process chosen in this study, an alcohol precursor is converted into the corresponding ester by a His_6_-tagged enzyme, immobilized on nitrilotriacetic acid (NTA) functionalized agarose beads inside a silicon-glass microreactor [14]. The merits of enzyme immobilization have been explored extensively in the last decades. An excellent overview on general enzyme immobilization strategies can be found in the book by Cao [15]. Out of the many possible immobilization approaches, many of which have also been implemented in microfluidic systems [16], [17], we have chosen the interaction of histidine with Ni^2+^ chelated in nitrilotriacetic acid (NTA). The polyhistidine tag is perhaps the most popular genetically encoded affinity tag [18], well-known for its facile application and reversibility of binding. After functionalization of a surface with NTA or iminodiacetic acid (IDA), treatment with a solution of a transition metal ion [19] renders the surface suitable for immobilization of a His_n_ tagged protein (where n can range from 6 to 12). This strategy is broadly implemented in immobilized metal affinity chromatography (IMAC) for reversible capture and purification of proteins, which can then be resolubilized with imidazole [20]. Despite the weak interaction between the His-tag and NTA/Ni^2+^, characterized by a desorption constant K_d_ in the micromolar range (1 to 10 µM), this immobilization approach has been applied to microfluidic and sensing systems, either by binding to beads packed in a microchannel [21] or directly to a glass substrate [22]. For simplicity, we have chosen to use a bead-based approach similar to what was described by Srinivasan et al. [21]. More details about the implementation of this approach, and more specifically on the pretreatment of microreactor inner walls to prevent adsorption of enzyme substrate and product, can be found in our previous publication [14].

Until recently, the release of chemical stimuli on insect flight behavior in e.g. wind tunnel experiments relied mainly on the passive evaporation of volatile chemicals from a lure, usually made from a filter paper or a rubber septum [23]. A key drawback of the approach is that only the initial dose applied to these lures is specified, while other factors such as chemical affinity to the substrate used, the kind and amount of solvent in application, temperature, airflow above the lure and time of evaporation are often overlooked. Therefore, establishing compound ratios and their emission rates is cumbersome, time consuming and poorly reproducible, especially for low concentrations of volatiles released from such traditional lures. As a solution to some of the key underlying problems, we have developed a micromachined evaporator which releases on demand a defined amount of pheromone into the atmosphere, by using a controlled flow of liquid into a heated, partially open compartment. Although not shown in this study, the device (or several devices in parallel) would allow a fast and timely definition of the content of evaporated plumes in terms of ratiometrical and temporal coding in future applications.

In the same class of devices the closest would be an evaporator with ultrasound. Even though ultrasonic devices allow to control the release of volatiles, it is often the case that insects are sensitive to the ultrasound emanating from the device. Some species respond to ultrasound within the working frequency range of the devices during mate orientation and courtship [24]–[26]. To circumvent such source-related side effects it is necessary to exchange the piezo in order to have the system adjusted to an insect species [27]. Therefore our microevaporator is based on controlling the vapor pressure above a pheromone solution [28] by temperature and the flow rate of the pheromone into the heated section of the device. Optionally, the air flow above the outlet of the device may be controlled by varying the amount of purging gas in the headspace. This process could eventually be automated.

The combination of a microreactor with immobilized *atf*, which produces the major component of the *S. Literalis* pheromone, and a microevaporator which releases a pre-defined amount of this component forms a biomimetic chemoemitter or *artificial gland*. The purpose of this paper is to demonstrate the functionality of this chemoemitter and its implementation for simultaneous pheromone synthesis, dissipation and later detection and/or quantification by male moths in behavioral and electroantennographic experiments.

## Materials and Methods

Branched polyethyleneimine (PEI) 50% (wt) solution with molecular weight 750 kDa polycation, and dextransulfate sodium salt (DSS) from Leuconostoc spp. as polyanion with molecular weight 500 kDa were used in the layer-by-layer deposition. The cross linking reagent, crotonaldehyde, sodium cyanoborohydride, sodium chloride (NaCl), acetate buffer, borate buffer, dodecyl acetate (97%), anhydrous toluene, N,N-dimethylformamide (DMF), dimethyl sulfoxide (DMSO), glycerol, acetyl coenzyme A sodium salt (acetyl-CoA), Trizma HCl (Tris-Cl), hydrogen peroxide (H_2_O_2_, 30%), (3-glycidoxypropyl)trimethoxysilane (97%) and sulfuric acid (H_2_SO_4_, 98%) were obtained from Sigma-Aldrich (Chemie BV, Germany), and used without further purification. (Z,E)-9,11-Tetradecadienyl acetate was purchased from Bedoukian (Danbury, USA) and (Z,E)-9,11-tetradecadien-1-ol was obtained from it by hydrolysis with KOH/ethanol.

The agarose beads were part of the His Band purification kit from Novagen (Darmstadt, Germany). One side polished 4-inch silicon (100) wafers were commercially available from Okmetic (Vantaa, Finland), and the 4-inch borofloat glass wafers were purchased from Schott AG (Benelux, Netherlands).

### Acetyl transferase

Although *atf* enzymes participate in the biosynthesis of many insect pheromones, to our knowledge no *atf* cDNA has been isolated from the pheromone gland of an insect. The absence of a reference sequence was an important handicap to identify new cDNA sequences, making our attempts to isolate *S. littoralis atf* cDNA unsuccessful. This scenario forced us to look for an alternative enzyme to be used for our purpose. Besides insect pheromones, transfer of an acyl group to an alcohol occurs in the biosynthesis of a variety of chemicals, such as neurotransmitters [29], plant volatiles [30] or waxes [31]. Among them, the previously identified wax synthase from *Acinetobacter sp.* [32] appeared to be a good candidate for our goal. This synthase can produce wax molecules from long chain alcohols (C_12_ to C_20_, a range covering the C_14_ chain of the pheromone alcohol of *S. littoralis*) and a long chain acyl derivative. The capacity of the enzyme to accept substrates structurally related to pheromone precursors and the simplicity of the recombinant expression system necessary to obtain the isolated enzyme led us to select the *Acynetobacter* wax synthase for our work.

The expression vector pET23a containing the wax synthase gene was kindly sent to us by Dr. Stefan Uthoff (Westfälische Wilhelms-Universität). The plasmid was transformed in *Escherichia coli* (expression strain RosettaTM(DE3)pLysS, Novagen) and expressed according to the vector manufacturer specifications. After expression of the protein, the cell culture was disrupted using a French press and clarified by centrifugation. The protein of interest was then purified by metal affinity chromatography with a nitrilotriacetic acid (NTA)-Ni^2+^ resin. The salts and other undesired compounds from the purification process were removed by gel filtration chromatography, and the sample (containing 10% of glycerol as cryoprotector) was concentrated and frozen in liquid nitrogen for storage. The whole process was optimized to obtain 130 µg of pure protein/100 mL of cell culture.

### Long-term activity assay with immobilized atf on beads

One hundred µL of suspension of NTA-functionalized agarose beads were introduced into Eppendorf tubes, washed and charged with Ni^2+^ according to the supplier's recommendation (Novagen, Darmstadt, Germany). Then 3 µg of His_6_-tagged diacylglycerol acyl transferase (*atf*) were added and incubated for 40 min in 10 mM Tris-Cl, pH 7.3, containing 10% glycerol in two consecutive steps. The beads were shortly washed with the same buffer, the liquid was decanted and the beads were transferred to glass vials (4 mL). To each vial 200 µL of the reaction mix were added and the vials were incubated for 0, 19, and 32 h at 35°C. A control experiment was set in parallel with the same amount of non-immobilized enzyme. The experiments were made in duplicate and the substrate concentration from the initial mix was measured by quantitative GC-MS after hexane extraction [14].

### Fabrication of the silicon/glass microreactor

Fabrication of the silicon-glass microreactor followed well established techniques previously described by us [14] with two variations consisting of a meandering channel with length (l = 9.8 mm) and a rectangular cross-section: the first microreactor (width = 250 µm, height = 50 µm), and the second microreactor (width = 300 µm, height = 50 µm) for longer retention times. Both variations were studied in activity assays and by numerical modeling (see below).

### Polyelectrolyte layer-by-layer deposition and His_6_-atf immobilization on NTA-agarose beads

Piranha (mixture of H_2_SO_4_∶H_2_O_2_ 3∶1, v/v) activated and dried microchannel surfaces were incubated with 1.5% (3-glycidoxypropyl)trimethoxysilane solution in anhydrous toluene [33] for 2 h and rinsed with DMF. The initial layer of the layer-by-layer deposition [34] was done with 0.01% (wt) PEI (750 kDa) from DMF, and the consecutive layers from 10 mM borate buffer solution at pH 9.2. Crotonaldehyde (4.1 µM) was added and the salt concentration was adjusted to 1.7 mM NaCl solution. The time of incubation in stopped flow with the PEI solution was 10 min at room temperature. The loosely bound PEI molecules were removed with sufficient amounts of Milli-Q water.

The microreactor was refilled with an aq. soln. containing 0.4% (wt) DSS in 10 mM acetate buffer, pH 3.8 and 1.7 mM NaCl, and incubated for 10 min at room temperature. Non-adsorbed DSS molecules were discarded by rinsing with Milli-Q water. A total of five layers were deposited alternating PEI with DSS to end the build-up with positively charged PEI. The distance between the outlet and the capillary was adjusted to prevent leakage of the beads. After the surface was dried with a N_2_ flow, 100 µL of suspension of NTA-functionalized agarose beads were introduced into the microreactor. Consecutively, the system was washed and charged with Ni^2+^ according to the supplier's protocol (Novagen, Darmstadt, Germany).

### Activity assay inside the microreactor with immobilized atf

The two microreactors were tested separately at various flow rates, and a fraction of the eluted product was collected (0.2 mL) from each one. The fractions were extracted with hexane and the contents of substrate and product was evaluated by GC-MS. The residence times τ (min) of the substrate and product were calculated from the volumetric flow rates as τ = V/F, where V is the volume of the microreactor and F is the volumetric flow rate, with the assumption that the rheological conditions inside the microreactor are close to a plug-flow regime.

### Numerical modeling

An ordinary differential equation based (ODE) model was constructed (Eqs. 1–3) and applied for characterization of *atf* after immobilization on agarose, in order to estimate the enzyme kinetics considering adsorption of substrate, product as well as enzyme deactivation in time.

**Figure 1:**
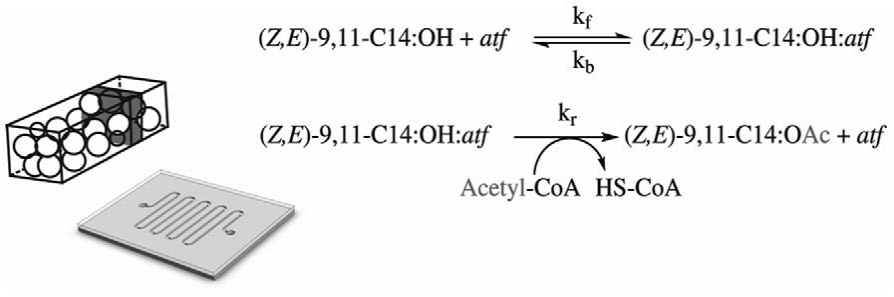
Drawing of the Silicon-glass microreactor with a section of the microchannel packed with NTA-functionalized agarose beads, with the dark grey box representing an infinitesimal volume *n*, together with the canonical enzymatic reaction, based on the Michaelis and Menten kinetics, adapted for substrate conversion with *atf*.

Figure 1 shows a schematic representation of the biomicroreactor used for production of the main pheromone acetate of *S. littoralis* from the substrate (*Z,E*)-9,11-C14:OH. The presented equation in Figure 1 for the main substrate (*Z,E*)-9,11-C14:OH was applied to characterize the *atf* kinetics in the microreactor system. At steady state d[ES]/dt = 0, where ES is the complex enzyme-substrate, the modeling is achieved by using mass balance equations (Eqs. 1–2) for substrate and product (see Information S1 for details) can be formulated as:
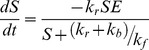
(1)

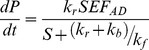
(2)In Eq. 2 a parameter (adsorption factor, F_AD_) has been introduced to account for the retention of product on the agarose beads. This factor value is derived from an experimental batch reaction using the enzyme immobilized on beads. Another important parameter was also estimated from the same set of experiments, the enzyme deactivation constant (K_D_), which denotes the deactivation rate of the immobilized *atf* with time. Its value was used in the equation as,
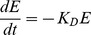
(3)In order to solve the system of differential equations (Eqs. 1–3) a number of experimental values were considered. The equations were integrated using free parameters (a free parameter is defined by Chen and coworkers [35] as a constant in a model that has no a priori value and must be estimated), for the forward, reverse and catalytic rate constants, in order to establish correlation of conversion with time. An overall description of the parameters used and their values is given in Table 1.

**Table 1:** Parameters used in the analytical solution of the ODE system.

Parameter description	Symbol	Value	Units
**Immobilized enzyme**	E	10	µg
**Substrate concentration** * **(initial)**	S_0_	119	µM
**Complex formation constant**	k_f_	free parameter	µM sec^−1^
**Complex dissociation constant**	k_b_	free parameter	sec^−1^
**Product formation constant**	k_r_	free parameter	sec^−1^
**Enzyme deactivation constant** **	K_D_	1.4	h^−1^
**Adsorption factor** *	F_AD_	0.34	—
**Product concentration (initial)**	P_0_	0	µM

* Determined from the amount of substrate after incubation of the reaction mixture with agarose beads without immobilized *atf*.

** Determined in a preliminary long-term activity assay of immobilized *atf* on beads in batch.

The system of ODEs was analytically solved in MatLab (Matlab 2008b, The MathWorks, US) by using a fourth-order Runge-Kutta method of integration. The program code is available in Information S1.

### Microevaporator

The evaporator consists of a silicon membrane (5.00×5.00×0.04 mm) perforated with ca. 40.000 micromachined via-holes. The device has integrated heaters and temperature sensing elements. Microfluidic channels deliver the mixture of predefined volatile compounds from two inlets to the reservoir located under the membrane, from which the mixture is completely evaporated by heating. Detailed information can be found in our previous conference paper [36], part of which has been included in the Information S1. The efficiency of the evaporator was evaluated by adsorption in Porapak cartridges of the vapor released from a 10 µg/µl solution of commercial (*Z,E*)-9,11-C14:OAc in hexane at different flow rates. In addition, the released pheromone was tested for activity in behavioral experiments. To this purpose, the evaporator was placed at the far end of a wind tunnel and *S. littoralis* males were released from a platform at the closer end [37]. A 10 ng/µl aqueous solution of the pheromone (*Z,E*)-9,11-14:OAc containing 4% DMSO was introduced into the system with a flow rate of 2 µl/min. The evaporator was heated to 120°C to ensure complete evaporation of the water. Failure to do it would cause an accumulation of the water+pheromone molecules inside the evaporator yielding unreliable results. In addition, trials to lower the temperature of the evaporator by using organic solutions of the pheromone in hexane or ethanol failed to evoke any insect response (see animal flight assays below).

### Wind tunnel experiments

Assays were conducted in a glass tunnel of 180×50×50 cm as previously described [38]. The wind was pushed through the tunnel by a 30 cm diameter fan at 20 cm/s. The tunnel was illuminated with two red light fluorescent tubes dimmed to 1 lux. The temperature was maintained at 25±2°C, and the relative humidity was 43±10%. Insect males were acclimatized to the experimental conditions of the tunnel for 30 min, and individually released into the tunnel between the 4th and the 6th hour into the scotophase. Before the tests, the insects were placed on filter paper in a Petri dish, and then introduced into the tunnel at a height of 20 cm and a distance of 125 cm from the emission source. After a 30 s acclimatization period, the behavior of males was recorded for 5 min. For each responding insect, the following four types of behavior were recorded: taking flight; halfway (arrival to the middle of the tunnel); close approach (flight within the plume to the surroundings of the pheromone) and source contact (contact with the pheromone source). Insects were subjected to the following attractant sources: a filter paper loaded with 10 µg of (*Z,E*)-9,11-tetradecadienyl acetate in hexane; 3 virgin females placed inside a cage exhibiting a calling behavior; the evaporator releasing a 10 ng/µl of an aq. solution of the pheromone containing 4% DMSO, or an aq. sol. containing only 4% DMSO (blank). For statistical analyses, a χ^2^ homogeneity test (P<0.05) was performed for every treatment.

### Electrophysiology

The EAG apparatus was commercially available from Syntech (Hilversum, The Netherlands). In brief, male antennae were excised, cut on both ends, and fixed to both electrodes with conducting gel Spectra 360 (Parker Lab. Inc., Hellendoorn, The Netherlands). A flow of humidified pure air (1000 ml/min) was directed continuously over the male antenna through the main branch of a glass tube (7 cm long×5 mm diameter). Test stimulations were carried out by giving puffs of air (300 ml/min) for 100 ms with the aid of a stimulus controller CS-01 (Syntech). Stimuli were applied at intervals of 30 sec on 10 antennae, and four times on each antenna. Control puffs with a piece of paper containing 10 µg of commercial (Z,E)-9,11-tetradecadienyl acetate were also intercalated between two consecutive stimuli to normalize the responses. The signals were amplified (100×) and filtered (DC to 1 kHz) with an IDAC-2 interface (Syntech), digitalized on a PC, and analyzed with the EAG Pro program. Depolarization means were compared for significance using analysis of variance (ANOVA) followed by LSD tests (P<0.05).

## Results and Discussion

### Enzyme activity after immobilization in microreactors

**Figure 2:**
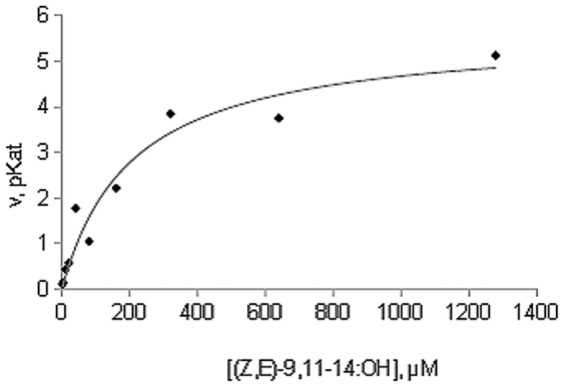
Plot of reaction velocity of *Acinetobacter* wax synthase vs substrate (*(Z,E)*-9,11-tetradecadienol) concentration (1.25 µM–1.3 mM) giving in batch a Michaelis-Menten profile.

Prior to immobilization of the *Acinetobacter* wax synthase in a microreactor, the activity of the enzyme on the pheromone precursor (*Z,E*)-9,11-14:OH was evaluated in batch. To this end, the enzyme was incubated in the presence of 60 µM of the alcohol and 300 µM of acetyl-CoA at different times. After verifying the linearity of the reaction after 25 min reaction, the enzyme was incubated in the presence of ten different substrate concentrations ranging from 1.25 µM to 1.3 mM. The reaction velocities were plotted *vs* substrate concentrations giving a Michaelis-Menten profile (Figure 2). The transformed Hanes-Woolf plot of [S]/v versus [S] gave a straight line (r^2^ = 0.8) from which the following kinetic constants of the enzyme in batch were determined: Km^ap^ 1.63 µM, Vmax^ap^ 5.5 pKat.

**Figure 3:**
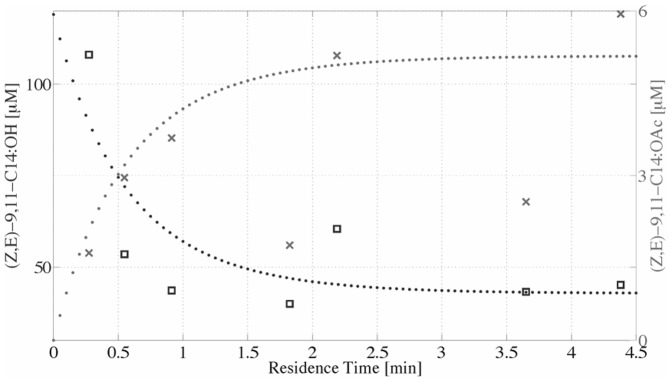
Scatter plot of conversion as a function of residence time in microreactor. Displayed are the concentrations of substrate (squares), product (crosses) recovered from two different microreactors with equal amounts of *atf* and the resulting (dashed) curves resulting from the numerical modeling. The experimental points show large scatter, probably due to uncontrolled loss of substrate or product in some experiments.

A scatter plot of the residence time inside the microreactor *vs.* the calculated concentration of the remaining alcohol and the estimated amount of the pheromone acetate yielded hyperbolic curves (Figure 3), resulting from the equations (1)–(3). The experimental values represented as squares (substrate) and crosses (product) in Figure 3 fit to the estimated curves. From these values the calculated V_max_ inside the microreactor resulted to be 19.4 pKat.

We have noticed that the enzyme has lost its activity 32 h after immobilization (results not shown) in contrast to the activity of the immobilized Lipozyme IM, which remained stable for more than 180 days of continuous working without losing catalytic activity [39]. It should be noticed, however, that although the *atf* has moderate stability after immobilization the margins of its activity are enough to allow short-term experiments, as those conducted here. Another factor that influences the yield of the pheromone is adsorption. Despite surface modification, adsorption is not completely prevented by the polyelectrolyte multilayer [14], the coating being responsible for 40% reduction of the pheromone produced. In the current model, we contemplate two causes for the adsorption of pheromone: interactions with the agarose carrier (F_AD_) and adsorption on the microreactor surface. However, these two factors do not completely explain the low yield of pheromone acetate from the microreactor. According to the literature, however, other causes may explain lower enzyme activity after immobilization, such as changes in protein conformation leading to steric hindrance of the active site [40], and/or presence of a diffusion layer around the support that would limit the mass-transfer in and out of the reaction zone [41].

### Evaporator efficiency

The efficiency of the evaporator was evaluated by adsorption of commercial pheromone in Porapak cartridges and behavior in wind tunnel experiments. In the first case, a linear correlation was established between the flow rates and the amount of recovered pheromone, which was higher than 80% of the theoretically released pheromone. For the purpose of the current study, the evaporation experiments were deliberately restricted to a single pheromone molecule and its precursor. However, other volatiles can also be evaporated in pre-defined amounts by varying the flow rates and/or temperature in a controlled manner. An advantage of our approach is that consumption of pheromone is significantly reduced due to the low volume (375 nL) of the evaporation chamber. Furthermore, the microevaporator has a high heat transfer, allowing it to reach the desired temperature very rapidly and maintaining it within 30 mK, if necessary. This fast heat response would allow a controlled release of pheromone into the air.

**Figure 4:**
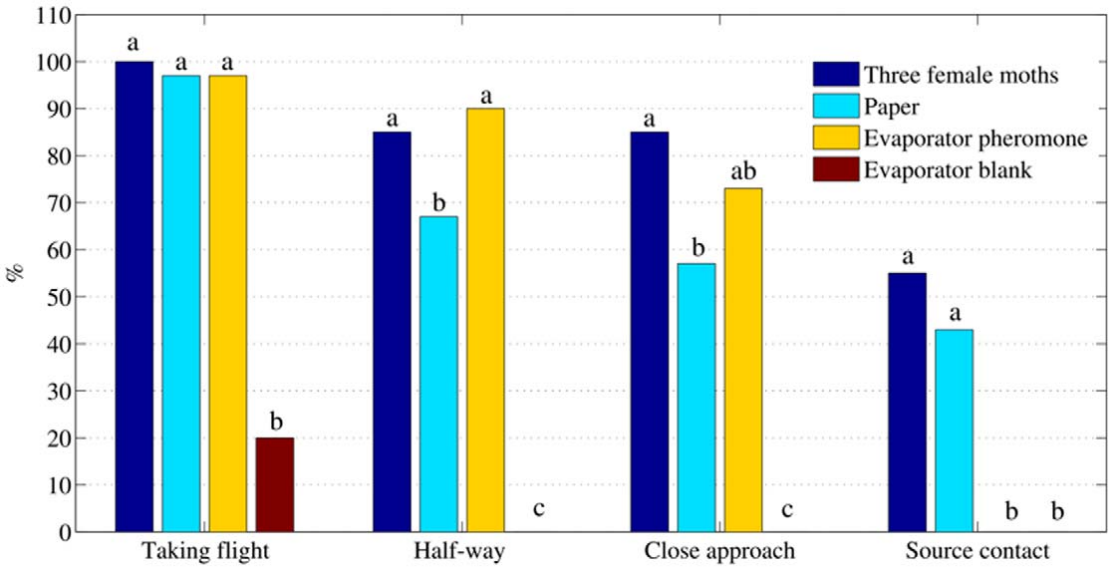
Behavioral responses of *S. littoralis* males (N = 20 for each assay) to a 10 ng/µl aq. solution of the pheromone containing 4% DMSO released by the evaporator (evaporator pheromone); 3 virgin females; a filter paper containing 10 µg of the pheromone in hexane and to the blank. Same letters over bars corresponding to the same behavior are not significantly different (χ^2^ test, P<0.05).

In wind tunnel assays, we compared the behavior of males responding to the pheromone delivered from the evaporator vs. other pheromone sources. The pheromone released from the evaporator elicited a similar attraction as 3 virgin females in most of the flight parameters considered (Fig. 4): 97% *vs.* 100% of insects taking flight, respectively; 90% *vs.* 85% of males arriving to the middle of the tunnel and 73% *vs.* 85% that closely approached to the source. Insects failed, however, to contact the source in the presence of the evaporator, most likely because of the high working temperature in its surroundings (120°C). In the blank experiment, only 4 out of 20 insects (20%) took off from the platform but none of them showed an attractive response nor followed an oriented flight.

A representative flight of an insect responding to the evaporator releasing pheromone was video-recorded (see Movie S1). In the video, the insect shows an oriented flight in the direction of the evaporator, until the thermal receptors of the moth prevent contact with the pheromone source. In blank experiments, in which the evaporator emitted only solvent and no pheromone, none of the tested insects showed attractant behavior.

### System integration: microreactors, evaporator and electroantennography

**Figure 5:**
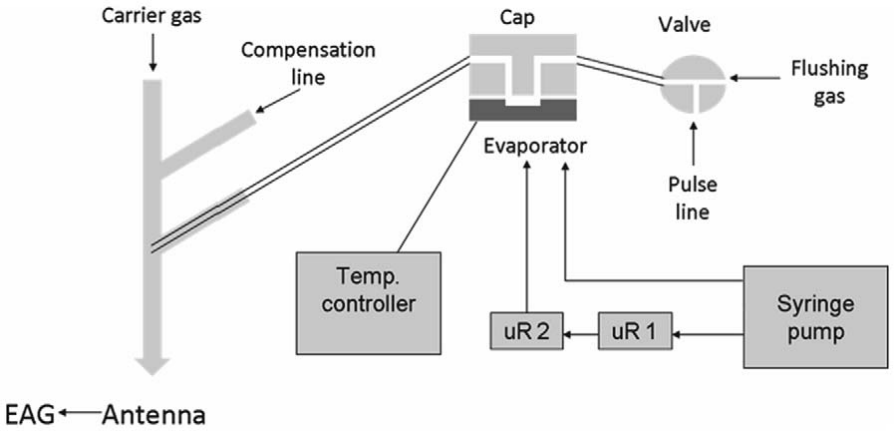
Schematic representation for the integration of two microreactors (uR1, uR2), evaporator and EAG detection.

The performance of the entire chemoemitter was assessed by electroantennography. To this purpose, two microreactors, evaporator and an EAG system were connected to produce, evaporate and detect the pheromone simultaneously (Fig. 5). The response to the pheromone emerging from the microreactors (connected in series to increase conversion) and the blank (320 µM aq. soln. of (*Z,E*)-9,11-14:OH containing 4% of DMSO), directly introduced into the evaporator from the syringe pump at a flow rate of 2 µl/min, was measured. The EAG recordings were normalized to the standard response elicited by 10 µg of synthetic (*Z,E*)-9,11-14:OAc deposited on a filter paper, which was recorded just before each experiment to avoid differences in response by different antennae or measurements at different lifetimes of the same antenna.

**Figure 6:**
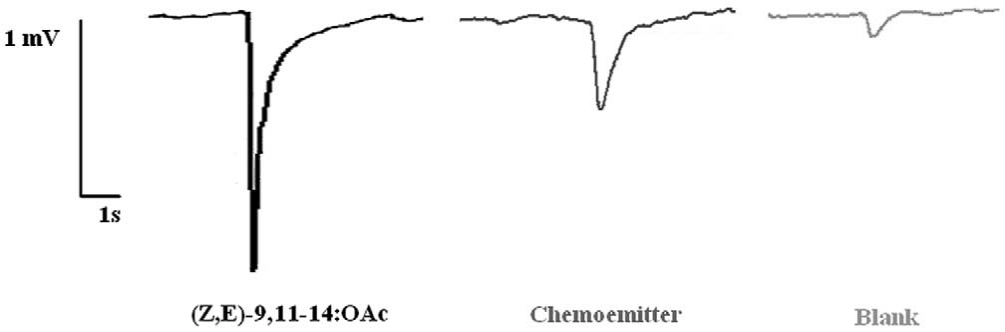
Electroantennographic detection of the pheromone produced by two microreactors and emitted by the evaporator (center) *vs* response to a filter paper containing 10 µg of the synthetic pheromone (left) and blank (right).

**Figure 7:**
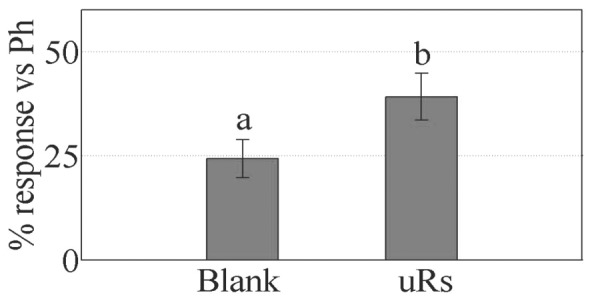
Mean percentage of the EAG response from 10 insect antennae to the blank and the pheromone released from the microreactors (“uRs”) relative to the response to 10 µg of synthetic pheromone.

Representative EAG traces of the synthetic pheromone (left), pheromone delivered by the chemoemitter (center) and blank (right) are displayed in Figure 6. Figure 7 [42] shows the mean percentage of the depolarization induced by the blank and by the pheromone emerging from the chemoemitter after partial conversion of (*Z,E*)-9,11-14:OH into (*Z,E*)-9,11-14:OAc *vs.* the response elicited by puffs of 10 µg of synthetic pheromone. The difference was found to be significant (Student *t* test, P<0.01) which proves the efficient production, release and detection of the pheromone by the integrated system.

To quantify the amount of pheromone produced and released from the chemoemitter, a calibration line (y = 1.0658x+3.3392; R^2^ = 0.9877) was created (see Information S1) by fitting the data of corrected EAG response (pheromone minus blank) vs. flow rate of a 10 ng/µl aq. solution of pheromone containing 4% DMSO (flow rate range: 0.01 to 2 µl/min). From this calibration it is derived that the mean corrected EAG response to the pheromone from the microreactor corresponds to a concentration of pheromone of approx. 5 ng/µl, which is consistent with the GC-MS analysis of an aliquot of the pheromone solution released from the two microreactors (3.2 ng/µl). Therefore, the EAG appears to be a suitable technique for detection and quantification of the pheromone produced by the chemoemitter system.

## Conclusions

We have developed a chemoemitter system, consisting of a microreactor and an evaporator, capable to infochemically produce pheromone acetate from its precursor alcohol catalyzed by a biosynthetic enzyme (alcohol acetyl transferase), and dissipate the mixture of both compounds in a controlled manner, thus mimicking one important process of the chemical communication in insects. The chemoemitter performance has been demonstrated by electroantennography and by the (positive) response of male moths. The integrated system of the chemoemitter with the chemoreceiver may form the basis of a new technological platform for the transmission of chemical messages.

## Supporting Information

Figure S1 **Micromachined pheromone evaporator.** Left: Photograph of the evaporator chip seen from the side of the microchannels for pheromone inlet. Right, top: SEM photographs of membrane. Right, bottom: Schematic cross-section of the evaporator.
(TIF)

Figure S2 **Evaporation rate of a solution of ZE-9,11-14:OAc in hexane versus the injection flow rate.** Data obtained from GC-MS measurements of the pheromone vapour adsorbed on Porapack column.
(TIF)

Figure S3 **Calibration curve of corrected EAG response to pheromone ((Z,E)-9,11-14:OAc) evaporated from a 10 ng/µl aq. solution.**
(TIF)

Information S1 **Supporting information, giving the details of the design and fabrication of the micromachined evaporator, calibration curve of corrected EAG response, mass balance equations for enzymatic conversion, and MatLab function for analytic solution of three ODEs using a 4th-order Runge-Kutta method.**
(DOC)

Movie S1 **Oriented flight of insect responding to pheromone released by the evaporator.**
(MP4)
