# Supplementary figures and images for: Mimicking Insect Communication: Release and Detection of Pheromone, Biosynthesized by an Alcohol Acetyl Transferase Immobilized in a Microreactor
Source: PLoS One. 2012 Nov 14;7(11):e47751. doi: 10.1371/journal.pone.0047751 (PMC3498290; doi:10.1371/journal.pone.0047751)

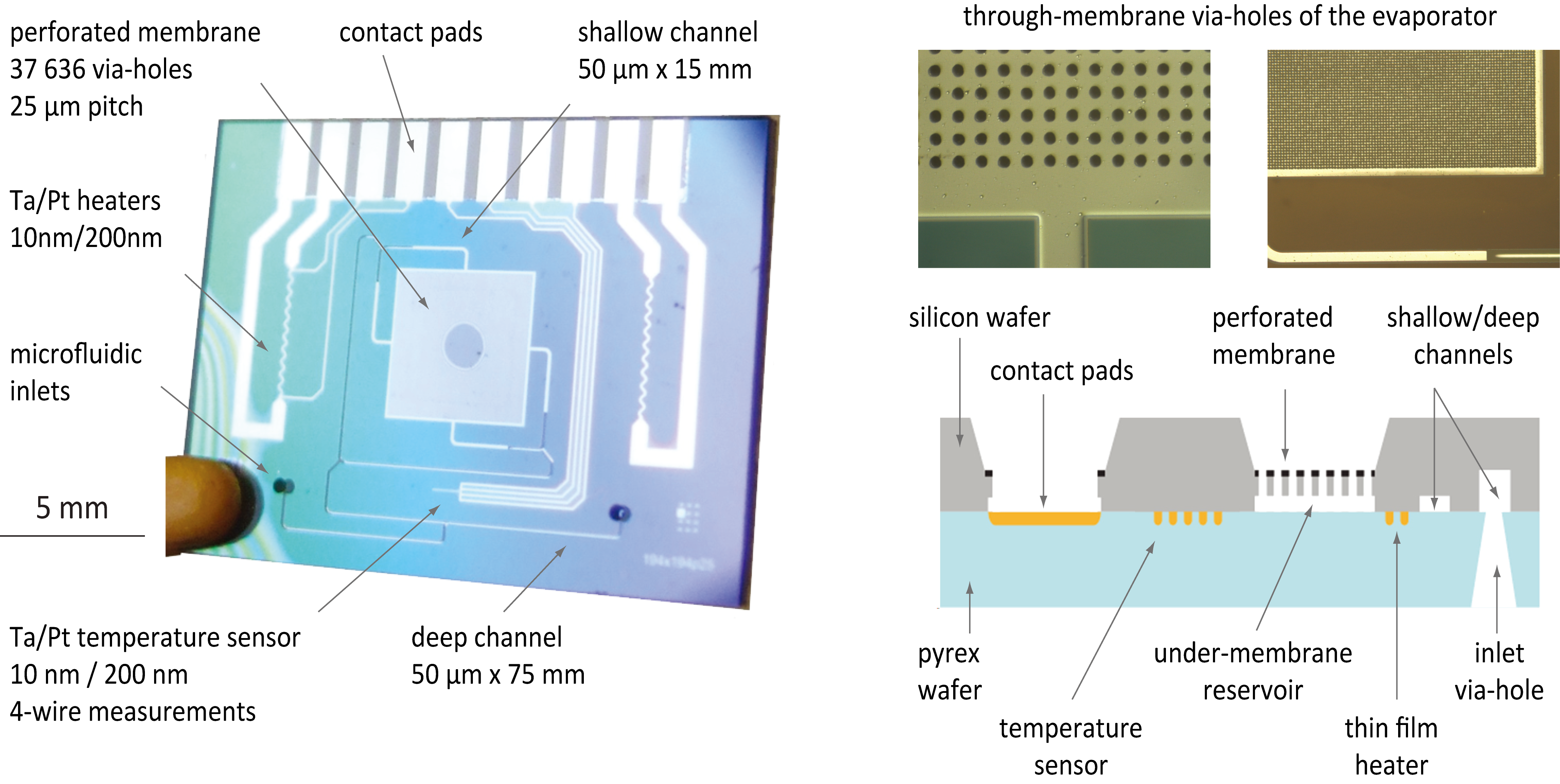

Supplement: Figure S1 — Micromachined pheromone evaporator. Left: Photograph of the evaporator chip seen from the side of the microchannels for pheromone inlet. Right, top: SEM photographs of membrane. Right, bottom: Schematic cross-section of the evaporator. (TIF) [file pone.0047751.s001.tif]

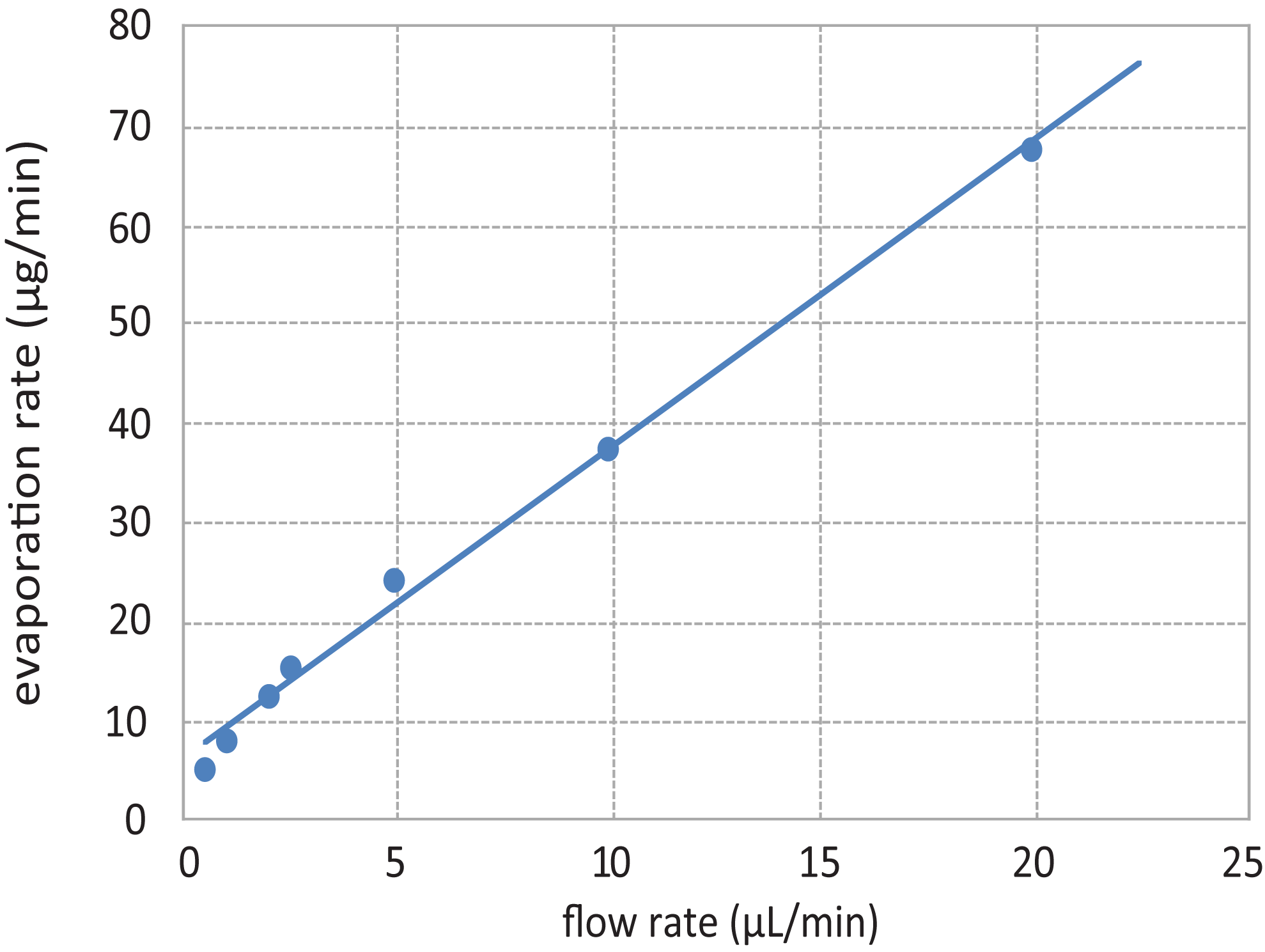

Supplement: Figure S2 — Evaporation rate of a solution of ZE-9,11-14:OAc in hexane versus the injection flow rate. Data obtained from GC-MS measurements of the pheromone vapour adsorbed on Porapack column. (TIF) [file pone.0047751.s002.tif]

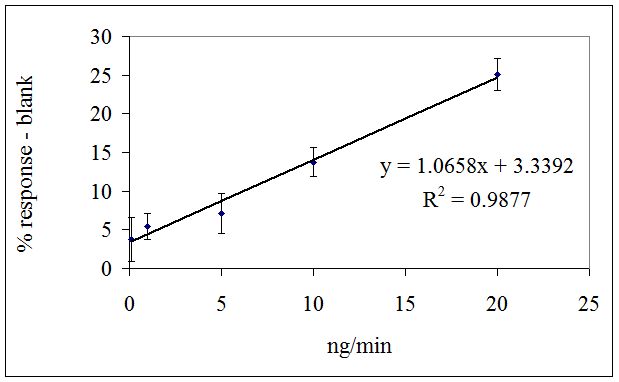

Supplement: Figure S3 — Calibration curve of corrected EAG response to pheromone ((Z,E)-9,11-14:OAc) evaporated from a 10 ng/µl aq. solution. (TIF) [file pone.0047751.s003.tif]
