# Supplementary material for: Mimicking Insect Communication: Release and Detection of Pheromone, Biosynthesized by an Alcohol Acetyl Transferase Immobilized in a Microreactor
Source: PLoS One. 2012 Nov 14;7(11):e47751. doi: 10.1371/journal.pone.0047751 (PMC3498290; doi:10.1371/journal.pone.0047751)
Supplement: Information S1 — Supporting information, giving the details of the design and fabrication of the micromachined evaporator, calibration curve of corrected EAG response, mass balance equations for enzymatic conversion, and MatLab function for analytic solution of three ODEs using a 4th-order Runge-Kutta method. (DOC) [file pone.0047751.s004.doc]

Mimicking insect communication: Development of a chemoemitter for pheromone biosynthesis by an alcohol acetyl transferase immobilised in a microreactor, release and detection

Lourdes Muñoz, Nikolay Dimov, Gerard Carot-Sans, Wojciech P. Bula,

Angel Guerrero and Han J. G. E. Gardeniers

**SUPPORTING INFORMATION**

**Micromachined evaporator (see also ref. SI-1)**

The microevaporator consists of a silicon membrane (5 mm x 5 mm x 40 μm) perforated with 37636 micromachined viaholes. Rectangular microfluidic channels deliver the mixture of predefined volatile compounds from two inlets to the reservoir (0.375 μL) located under the membrane, see Fig. S1.

The liquid passes through the holes in the membrane and evaporates from small droplets that are formed on the outlet of every hole. Two thin-film platinum heaters and a 4-wire resistive temperature sensor are integrated in the evaporator and are operated in a PID loop in order to stabilize the temperature with a maximum variation of 30 mK. The heaters can dissipate up to 8W of energy, which raises the temperature of the membrane up to 250°C.

The performance of the device for pheromone evaporation was tested with the aid of a dynamic headspace technique, in which the evaporator was setup in such a way that the pheromone vapour could be trapped on the column material in a Porapak cartridge. To this end, a PMMA cover was attached with its gas inlet and outlet via a PDMS pad to the membrane side of the chip. The temperature was set to 120°C and a solution of Z,E-9,11-14:OAC (10 μg/μL in hexane) was delivered to the chip by a syringe pump controlled at flow rates in the range 0.5 - 20 μL/min. The gas flow rate through the headspace was set to 250 mL/min, and the collection time varied from 7.5 to 60 min. After this, the Porapak cartridge was extracted with hexane, after which the pheromone content in the hexane was quantified by gas chromatography ciupled to mass spectrometry (GC-MS). The resulting data are shown in Figure S2, which exhibits a linear relationship between the evaporation rate and the inlet pheromone flow rate. With this calibration curve it is possible to establish a desired pheromone evaporation rate.

**Calibration curve**

A calibration curve of corrected EAG responses (pheromone minus blank) to the pheromone ((Z,E)-9,11-14:OAc) evaporated from a 10 ng/µl aq. solution containing 4% DMSO at different flow rates (0.01-2 µl/min) is shown in Figure S3.

**Derivation of the mass balance equations for the substrate and product**

Equation (1) is the material balance of the substrate for segment *n*, which is the accumulation of substrate or the ingoing mass of substrate at position *n*-1 equals the sum of the outgoing mass of substrate at position *n* and the substrate converted in product.

$\Delta V\frac{dS_{n}}{dt}=S_{n-1}F_{n-1}-S_{n}F_{n}+r_{S_{n}}\Delta V$ (1)

Assuming that the density of the reaction mixture is constant (1) can be transformed by dividing both sides by ∆V,

$\frac{dS_{n}}{dt}=\frac{-\Delta\left(S\cdot F\right)}{\Delta V}+r_{S_{n}}$ (2)

By substitution of ∆V by W H ∆z and ∆S by dS we derive the partial differential equation as

$\frac{\partial S}{\partial t}=\frac{-1}{W\cdot H}\frac{\partial\left(S\cdot F\right)}{\partial z}+r_{S}$ (3)

In the current study the volumetric flow F is constant at each time point. Then

$\frac{\partial S}{\partial t}=\frac{-F}{W\cdot H}\frac{\partial S}{\partial z}+r_{S}$ (4)

To model the dynamics by simulation methods, the partial differential equation (1) could be written also as

$\frac{\partial S_{n}}{\partial t}=\left(S_{n-1}-S_{n}\right)\frac{\Delta V}{F_{n}}+r_{S_{n}}$ (5)

the expression ∆V/F_n_ = τ, the residence time of the liquid in a single segment. From the expression (4), assuming that ∂[ES]/∂t = 0, where [ES] stands for enzyme-substrate complex, the steady state equation is derived,

$0=-v\frac{dS}{dz}+r_{S}$ (6)

which means that the rate of substrate depletion due to product formation with the flow along the channel equals the rate of the enzymatic reaction rS . For the infinitesimal volume, rSn is defined by the Michaelis-Menten kinetics as

$r_{S_{n}}=V_{m}S_{n}K_{M}+S_{n}$ (7)

Accordingly, the mass balance equations for substrate and product can be formulated as

$\frac{dS}{dt}=\frac{-k_{r}SE}{S+\frac{\left(k_{r}+k_{b}\right)}{k_{f}}}$ (8)

$\frac{dP}{dt}=\frac{k_{r}SEF_{AD}}{S+\frac{\left(k_{r}+k_{b}\right)}{k_{f}}}$ (9)

Enzyme deactivation is described as:

$\frac{dE}{dt}=-K_{D}E$ (10)

**MatLab function for solving analytically a system of three ODEs, by using a fourth-order Runge-Kutta method of integration**

function [t,x,y,z] = Sys3ODEsRK4(a, b, h, x1, y1, z1)

%Sys3ODEsRK4 will solve a system of three first-order initial-value ODEs using fourth-order Runge-Kutta method.

%The independent variable is t, and the dependent variables are x, y and z

prompt={'Enter the Kf:',...

'Enter the Kb:',...

'Enter the Kr'};

name='Input free parameters';

numlines=1;

defaultanswer={'0.15', '4.3', '20'};

%defines the initial values

answer=inputdlg(prompt,name,numlines,defaultanswer);

%allows to change the variables through "user-friendly interface"

global Kf; Kf = str2double(answer{1});fprintf('Kf= %5.4f\n',Kf);

%the variables resulting from the input dialog are transformed from str2double.

global Kb; Kb = str2double(answer{2});fprintf('Kb= %5.4f\n',Kb);

%IMPORTANTLY, they must be declared as global variables so they could be reached

global Kr; Kr = str2double(answer{3});fprintf('Kr= %5.4f\n',Kr);

% from a function from not nested in the main function (outside function).

global Fad; Fad = 0.12;fprintf('Fad= %5.4f\n',Fad); %Coefficients determined from the experimental data: for the adsorption, Fad;

global Kdeg; Kdeg = 1.05;fprintf('Kdeg= %5.4f\n',Kdeg);

% E deactivation, Kdeg.

ODE1 = @SubstrateDienol; %handle of the function Substrate Dienol with a name ODE1

function dxdt = SubstrateDienol(t,x,y,z)

% defines the function with its input arguments the same as for the function Sys3ODEsRK4

dxdt = - (Kr*x*(Kdeg*z))/((Kb+Kr)/Kf + x);

%relates the diff eq. with the function

end

ODE2 = @(t,x,y,z) Fad*(Kr*x*(Kdeg*z))/((Kb+Kr)/Kf + x);

%another option is to use anonymous function to describe the diff. eq.

ODE3 = @(t,x,y,z) -Kdeg*z;

t(1) = a; x(1) = x1; y(1) = y1; z(1) = z1;

n = (b - a)/h;

for i = 1:n

t(i+1) = t(i) + h;

tm = t(i) + h/2;

Kx1 = ODE1(t(i), x(i), y(i), z(i));

Ky1 = ODE2(t(i), x(i), y(i), z(i));

Kz1 = ODE3(t(i), x(i), y(i), z(i));

Kx2 = ODE1(tm, x(i)+Kx1*h/2, y(i)+Ky1*h/2, z(i)+Kz1*h/2);

Ky2 = ODE2(tm, x(i)+Kx1*h/2, y(i)+Ky1*h/2, z(i)+Kz1*h/2);

Kz2 = ODE3(tm, x(i)+Kx1*h/2, y(i)+Ky1*h/2, z(i)+Kz1*h/2);

Kx3 = ODE1(tm, x(i)+Kx2*h/2, y(i)+Ky2*h/2, z(i)+Kz2*h/2);

Ky3 = ODE2(tm, x(i)+Kx2*h/2, y(i)+Ky2*h/2, z(i)+Kz2*h/2);

Kz3 = ODE3(tm, x(i)+Kx2*h/2, y(i)+Ky2*h/2, z(i)+Kz2*h/2);

Kx4 = ODE1(tm, x(i)+Kx3*h/2, y(i)+Ky3*h/2, z(i)+Kz3*h/2);

Ky4 = ODE2(tm, x(i)+Kx3*h/2, y(i)+Ky3*h/2, z(i)+Kz3*h/2);

Kz4 = ODE3(tm, x(i)+Kx3*h/2, y(i)+Ky3*h/2, z(i)+Kz3*h/2);

x(i+1) = x(i) + (Kx1 + 2*Kx2 + 2*Kx3 + Kx4)*h/6;

y(i+1) = y(i) + (Ky1 + 2*Ky2 + 2*Ky3 + Ky4)*h/6;

z(i+1) = z(i) + (Kz1 + 2*Kz2 + 2*Kz3 + Kz4)*h/6;

end

end

**References**

SI-1 Bula WP, Dimov N, Muñoz L, Guerrero A, Gardeniers JGE (2010) Artificial gland for precise release of semiochemicals for chemical communication. Proc. 14th Int. Conf. Miniaturized Systems for Chemistry and Life Sciences (µTAS), Oct. 3-7, 2010, Groningen, The Netherlands, pp. 671-673.

**Legends to figures**

S1 Left: Photograph of the evaporator chip seen from the side of the microchannels for pheromone inlet. Right, top: SEM photographs of membrane. Right, bottom: Schematic cross-section of the evaporator.

S2 Evaporation rate of a solution of ZE-9,11-14:OAc in hexane versus the injection flow rate. Data obtained from GC-MS measurements of the pheromone vapour adsorbed on Porapack column.

S3 Calibration curve of corrected EAG response to pheromone ((Z,E)-9,11-14:OAc) evaporated from a 10 ng/µl aq. solution.
